# Person-centred Approaches to Psychopathology in the ABCD Study: Phenotypes and Neurocognitive Correlates

**DOI:** 10.1007/s10802-023-01065-w

**Published:** 2023-04-29

**Authors:** Chris Retzler, Glyn Hallam, Samantha Johnson, Jenny Retzler

**Affiliations:** 1 Department of Psychology, School of Human and Health Sciences, University of Huddersfield, Huddersfield, UK; 2 Department of Health Sciences, University of Leicester, Leicester, UK

**Keywords:** Latent class, Psychopathology, Preterm behavioural phenotype, Neurocognitive predictors

## Abstract

Issues with classifying psychopathology using narrow diagnostic categories have prompted calls for the use of dimensional approaches. Yet questions remain about how closely dimensional approaches reflect the way symptoms cluster in individuals, whether known risk factors (e.g. preterm birth) produce distinct symptom phenotypes, and whether profiles reflecting symptom clusters are associated with neurocognitive factors. To identify distinct profiles of psychopathology, latent class analysis was applied to the syndrome scales of the parent-reported Child Behaviour Checklist for 11,381 9- and 10- year-olds from the Adolescent Brain Cognitive Development study. Four classes were identified, reflecting different profiles, to which children were assigned probabilistically; Class 1 (88.6%) reflected optimal functioning; Class 2 (7.1%), predominantly internalising; Class 3 (2.4%), predominantly externalising; and Class 4 (1.9%), universal difficulties. To investigate the presence of a possible preterm behavioural phenotype, the proportion of participants allocated to each class was cross-tabulated with gestational age category. No profile was specific to preterm birth. Finally, to assess the neurocognitive factors associated with class membership, elastic net regressions were conducted revealing a relatively distinct set of neurocognitive factors associated with each class. Findings support the use of large datasets to identify psychopathological profiles, explore phenotypes, and identify associated neurocognitive factors.

**Supplementary Information:**

The online version contains supplementary material available at 10.1007/s10802-023-01065-w.

## Introduction

More than half of adolescents with a mental health problem meet diagnostic criteria for more than one disorder (Kessler et al., 2012), highlighting problems with classifying psychopathology using narrow diagnostic categories. This issue is recognised in the DSM-5 (American Psychiatric Association, 2013), which calls for further research into empirically-supported frameworks that allow a conceptualisation of psychopathology along broader dimensions. Dimensions such as internalising (a propensity to experience anxious, depressive and somatic symptoms) and externalising (a propensity to experience aggressive, impulsive and disruptive behaviour; Achenbach 1966; Achenbach et al., 2016) problems provide alternative ways to understand, diagnose and manage psychopathological difficulties. While dimensional approaches are not new, there is a need for greater knowledge of how such dimensions manifest in the population, whether they truly reflect the way symptoms cluster in individuals, and whether they form distinct or overlapping profiles of psychopathology. Large accessible datasets, such as the Adolescent Brain Cognitive Development (ABCD) study, provide new opportunities to identify subgroups of individuals with shared psychopathological profiles, and to explore ‘behavioural phenotypes’ thought to be associated with known risk factors. The comprehensive cognitive and neural data collected in the ABCD study also allows exploration of the neurocognitive factors that are associated with different psychopathological profiles (Dick et al., 2021).

One such behavioural phenotype is the ‘preterm behavioural phenotype’ (PBP) which has been associated with Very Preterm (≤ 32 weeks of gestation) birth. Among Very Preterm cohorts, there is an increased risk for inattention, anxiety and depression, and peer relationship difficulties relative to birth at term, with the risk for conduct problems or aggressive or delinquent behaviours remaining similar to term-born peers (Fitzallen et al., 2020; Hille et al., 2001; Johnson & Marlow, 2011; Mathewson et al., 2017; Wolke et al., 2019). This pattern of difficulties is echoed in diagnostic studies in which attention-deficit/hyperactivity disorder (ADHD), autism spectrum disorder (ASD) and anxiety and depressive disorders are the most prevalent psychiatric disorders after Very Preterm birth (Wolke et al., 2019). It is proposed that risk for this profile of symptoms results from interruptions to maturational processes in brain development or brain injury following Very Preterm or Extremely Preterm birth (≤ 28 weeks gestation; Volpe 2009), the risk of which increases as gestational age at birth decreases (Rogers et al., 2018). The PBP was proposed on the basis of diagnoses observed at the group level, and much of the evidence focuses on group-level analysis of symptoms common in cohorts born Very Preterm or Extremely Preterm. More research is required to better understand how behavioural symptoms cluster in individuals born preterm, whether those born Moderate-Late Preterm ( 32 to 36 weeks gestation) may be at risk for the PBP, and the extent to which this phenotype is unique to preterm birth.

Investigations of how dimensions of psychopathology are observed in the ABCD dataset more generally have, to date, only been conducted using forms of factor analysis (Michelini et al., 2019; Moore et al., 2020); a variable-centred technique. Variable-centred approaches can identify how symptoms align along dimensions in a dataset, but assume they align in the same way across all individuals in the population. Conversely, person-centred approaches to psychopathology assume that associations between symptoms can differ across individuals, and examine this heterogeneity to define subgroups for whom symptoms cluster in ways that are maximally similar within the group, and are different to individuals in other groups. Thus, person-centred approaches are a valuable way to investigate phenotypes and characterise psychopathological profiles.

In other datasets, researchers have demonstrated how applying person-centred approaches such as latent class analysis (LCA; Bianchi et al., 2017; Essau & de la Torre-Luque, 2019) and latent profile analysis (LPA; Basten et al., 2013; Bonadio et al., 2016; Webb et al., 2021) to dimensional measures in child and adolescent samples can help us better understand how symptoms cluster. Measures of psychopathology have included parent-report questionnaires such as the child behaviour checklist (CBCL; Basten et al., 2012; Bianchi et al., 2017), interviews such as the Composite International Diagnostic Instrument (CIDI; Essau & de la Torre-Luque, 2019), or multi-source measures (Bonadio et al., 2016; Webb et al., 2021). Samples have differed in (i) size, from smaller samples of 1,206 (Bonadio et al., 2016) to large samples of 10,123 (Essau & de la Torre-Luque, 2019); (ii) age, with some recruiting children only (Basten et al., 2012), adolescents only (Webb et al., 2021; Essau & de la Torre-Luque, 2019), or spanning childhood and adolescence (Bianchi et al., 2017; Bonadio et al., 2016); and (iii) source, with recruitment from the community (Basten et al., 2012; Webb et al., 2021; Essau & de la Torre-Luque, 2019) and clinically referred populations (Bianchi et al., 2017; Bonadio et al., 2016). Despite diversity in approaches, there is much consistency in the findings across studies.

Along with a ‘normative’ profile of individuals who display low or no risk for psychopathology, studies find a profile consistent with the internalising dimension of psychopathology, predominantly characterised by self-directed negative emotions such as anxiety and depression (Basten et al., 2013; Bianchi et al., 2017; Bonadio et al., 2016; Essau & de la Torre-Luque, 2019; Webb et al., 2021). Profiles aligned with the externalising dimension are more variable. Basten et al. (2013) and Essau & de la Torre-Luque (2019) identified a profile consistent with externalising problems, while Bonadio et al. (2016) identified *two* profiles characterised by externalising behaviour; one in which aggressive and oppositional behaviours were moderate, and the other distinguished by additional severe problems with delinquency and for which aggressive and oppositional behaviours were also more severe. On the other hand, rather than an externalising profile, Bianchi et al. (2017) identified a group with higher risk of inattention and hyperactivity, while the risk for symptoms of aggression and delinquency remained moderate and the risk for internalising problems was low. Finally, with the exception of Essau & de la Torre-Luque (2019), who found their sample was best described by only 3 profiles (normative, internalising, externalising), most studies have also identified a profile characterised by difficulties in most, if not all, domains (Basten et al., 2013; Bianchi et al., 2017; Bonadio et al., 2016; Webb et al., 2021). This is often termed the ‘dysregulation’ profile. Importantly, these profiles often do not map directly on to traditional diagnoses. For example, probabilities of diagnoses of ADHD are elevated to a similar degree in both internalising and externalising profiles (Essau & de la Torre-Luque, 2019). The use of person-centred approaches may therefore provide an important adjunct to more traditional diagnostic approaches and opportunities to enhance our mechanistic understanding.

Only a small number of studies have used person-centred approaches to examine the PBP. These studies include samples at a range of ages and born at a range of gestations; 8-year-olds born at < 28 weeks (Burnett et al., 2019), 5-year-olds born at < 30 weeks (Lean et al., 2019), 6-year-olds born at < 36 weeks (Poehlmann-Tynan et al., 2015), and 2-year-olds born at 32 to 36 weeks (Johnson et al., 2018) of gestation. Children were classified on the basis of the profile of behavioural and emotional difficulties they demonstrated (Burnett et al., 2019), but a number of studies also incorporated measures of cognition (Johnson et al., 2018; Lean et al., 2019; Poehlmann-Tynan et al., 2015) into the indicators used to classify subgroups. These studies showed that in infancy and childhood those born at preterm gestations were either over-represented compared with term-born children in profiles reflecting sub-optimal outcomes (Burnett et al., 2019; Johnson et al., 2018; Lean et al., 2019), or in the case of Poelhmann and colleagues (2015) which did not include a term-born group, the majority of the sample (70%) were allocated to sub-optimal classes. Consistent with the conception of the PBP informed by cohort studies, the sub-optimal profiles in which preterm-born children were over-represented emphasised the risk for elevated, but often sub-clinical difficulties (Lean et al., 2019), and with a tendency for higher risk of inattention and hyperactivity (Burnett et al., 2019; Lean et al., 2019), socio-emotional difficulties (Burnett et al., 2019; Johnson et al., 2018; Lean et al., 2019), and a lower risk of conduct problems (Burnett et al., 2019; Lean et al., 2019; Johnson et al., 2018). However, there is limited evidence of a profile that reflects a single set of difficulties specific to preterm-born individuals. Lean et al. (2019) identified a ‘school-based hyperactive-inattentive profile’ to which only 3% of term-born children, relative to 15% of Very Preterm children, were allocated, that they considered may reflect the PBP. Yet only Johnson et al. (2018), which recruited a large sample of children born Moderate-Late Preterm, identified a profile that was uniquely observed in their preterm sample, of which 7% were allocated to this class. Indeed, some studies found that Very Preterm (Lean et al., 2019) or Extremely Preterm (Burnett et al., 2019) children were over-represented in multiple sub-optimal classes, rather than a single class representing the PBP.

However, person-centred approaches such as LCA require large sample sizes for good class recovery, with an evidence-based heuristic indicating that at least 500 cases are required for most models (Nylund-Gibson & Choi, 2018). This requirement becomes more important still when subgroups of interest may comprise a small proportion of the overall sample (Nylund et al., 2007), particularly for researchers who wish to explore correlates of class membership. With the exception of Johnson et al. (2018; 638 Moderate-Late Preterm and 765 term), in previous studies using these approaches to examine the PBP fewer than 500 cases were included, with as few as 125 (85 Very Preterm) children included in Lean et al. (2019). Moreover, studies of the PBP have, to date, focussed on profiles observed in children aged 8 years or younger and there is a relative paucity of research into outcomes in adolescence.

Beyond preterm populations, person-centred studies of psychopathology in adolescence have also been limited in the extent to which they examine risk factors associated with sub-optimal profiles. This likely stems from the reliance on survey-based data collection to recruit large samples. Risk factors previously examined have included those that can be easily measured by self-report, such as demographics (e.g. Essau & de la Torre-Luque, 2019) or exposure to life experiences (e.g. Webb et al., 2021). Yet neurocognitive markers, which have been a common focus in studies of diagnostic groups, have not to our knowledge been investigated in relation to transdiagnostic profiles derived from person-centred analyses. Datasets such as those created by the ABCD study provide new opportunities to examine such associations.

Indeed, emerging work has begun to examine cognitive and neural correlates of latent dimensions of psychopathology identified via variable-centred approaches. For example, in the ABCD sample, general and specific dimensions of psychopathology have been linked to measures of broad cognitive function such as crystallised and fluid intelligence (Michelini et al., 2019), as well as more specific areas of cognition such as executive function (Cardenas-Iniguez et al., 2020; Romer & Pizzagalli, 2021) and indirectly (via executive functioning) to white matter microstructure (Cardenas-Iniguez et al., 2020). Executive function refers to the set of processes responsible for planning actions and regulating behaviour, including working memory and inhibitory control, which continue to mature through adolescence and have been linked to a variety of psychological disorders (Snyder et al., 2015). White matter microstructure, which reflects the structural integrity of white matter connections in the brain, has also been linked directly to general psychopathological risk in other samples (Neumann et al., 2020; Riem et al., 2019; Vanes et al., 2020).

Not only are executive functioning and white matter microstructure of particular interest when it comes to psychopathological risk, but both have been considered of mechanistic importance in relation to increased risk of psychopathology in preterm samples. A composite measure of executive function has been found to mediate the relationship between Very Preterm birth and total behavioural difficulties at school age (Schnider et al., 2020), while studies have also examined the interplay between specific executive functions and symptom domains (e.g. Retzler et al., 2019), showing that similar executive processes are implicated in both Very Preterm and term-born children. Similarly, white matter microstructure has been both pinpointed as a valuable transdiagnostic marker of psychopathology across the lifespan (Alnæs et al., 2018), but also specifically, white matter development is commonly adversely affected following preterm birth and has been associated with the PBP (Brenner et al., 2021; Loe et al., 2013). Research into the risk factors associated not with individual dimensions of psychopathology, but with the transdiagnostic profiles of symptoms that are actually experienced, is needed to further understand the neurocognitive correlates of behavioural difficulties, and the extent to which preterm birth may confer risk for specific psychopathological difficulties.

### The Current Study

The ABCD study recruited more than 11,000 pre-adolescents aged 9 to 10 years (Barch et al., 2018; Volkow et al., 2018). The data obtained at baseline included psychopathological, cognitive and MRI data, as well as retrospective parent-report measures of developmental history, including gestational age at birth. Although the sample was not recruited as a representative cohort of pre-adolescents born preterm, the numbers recruited provide a large sample in which to use person-centred approaches to examine profiles of psychopathology among pre-adolescents born preterm, and consider whether these reflect a PBP.

In the current analysis we made use of this comprehensive dataset to achieve three key aims. Firstly, we used LCA to identify separable classes based on psychopathology and allocate individuals to their most likely class (or psychopathological profile). Secondly, to ascertain whether a class that could reflect the PBP was observable, we tested whether preterm birth was associated with class membership. Finally, to build on evidence of relationships between neurocognitive factors and dimensions of psychopathology, we examined neurocognitive factors associated with class membership. Because cognitive factors beyond executive functioning may relate to dimensions and profiles of psychopathology (Blanken et al., 2017), measures of language, memory and processing speed included in the ABCD cognition test battery were analysed in addition to measures of executive function. From the range of neural markers available in the ABCD dataset, white matter microstructure was selected for this analysis based on evidence of its relevance to psychopathology.

## Methods

### Sample Recruitment and Selection

Details of the recruitment of participants to the ABCD cohort have been described in full elsewhere (Garavan et al., 2018). In brief, the ABCD study is a longitudinal study of neurocognitive development that aims to follow pre-adolescents for 10 years, from the age of 9 or 10 until they are aged 19 or 20. Probabilistic sampling methods were used to recruit a population-based and demographically diverse sample (Compton et al., 2019) of over 11,000 9- and 10-year-olds (between 2016 and 2018) from schools around 21 research sites in the USA. Schools were selected on the basis of urbanicity and the composition of the student population in terms of gender, race, ethnicity and socioeconomic status (Garavan et al., 2018). Although twins were recruited from four sites to facilitate studies of heritability, 81% of the sample analysed were singleton births (see Table 1). Written, informed consent and assent were obtained from parent or guardian and the child respectively prior to data collection. Baseline assessments, including retrospective parent-report measures of developmental history, were collected at the first visit when participants were aged 9 to 10 years. Follow-up assessments are ongoing and due to be conducted each year throughout adolescence.

For this study, baseline data for 11,878 participants were downloaded from the ABCD Study data repository (NIMH data archive; nda.nih.gov; curated release 3.0). A total of 497 participants were excluded from all analyses, with 331 excluded due to information on the screening questionnaire that indicated the presence of conditions that may affect their ability to complete the ABCD test batteries, or preclude the measurement of neurocognitive processing (namely, the presence of tumour, stroke, aneurysm, haemorrhage, hematoma, other medical condition), and 166 due to missing CBCL or gestational age data. Subsequent analyses included all participants with available data for the variables included in the analyses. The ABCD study received ethical approval from the University of California Institutional Review Board and our secondary analysis was approved by the University of Huddersfield. A record of the NDA study created in relation to this publication can be obtained using this DOI: 10.15154/1528202 (*to be released upon publication*).

### Measures

#### Symptomatology

Symptomatology at age 9 to 10 was measured using the parent-rated CBCL (age 6–18 version; Achenbach & Rescorla 2000) syndrome scales. Widely used in research and clinical practice to identify ‘problem’ behaviour in children, the CBCL has been shown to have good test-retest reliability (Achenbach & Rescorla, 2000), and has been validated as appropriate for understanding both narrow- and broad- band dimensions of psychopathology (Achenbach et al., 2016). The CBCL syndrome scales measure problems in eight dimensions; withdrawn, somatic, anxious/depressed, social, thought, attention, delinquent and aggressive. Items are scored as either 0 (not true as far as you know), 1 (somewhat or sometimes true), or 2 (very true or often true). For each scale we created a dichotomous variable, ‘elevated’ or ‘not elevated’, using the ‘borderline’ cut off on the T scores (scores ≥ 65 for all scales; Achenbach & Rescorla 2000).

#### Sample Characteristics

Sample characteristics were derived from ABCD study questionnaires completed by parents at baseline (i.e. when their child was aged 9 to 10).

Gestational age at birth was reported by parents as part of the developmental history questionnaire in an item that asked how many weeks before their due date their child was born. For analysis, this measure was used both continuously (converted into gestational age at birth in weeks), and categorically. Gestational age categories were defined in line with the World Health Organization (WHO, 2021): full term (≥ 37 weeks’ gestation; reported as born 0 to 3 weeks early); Moderate-Late Preterm (32 to 36 weeks’ gestation; reported as born 4 to 8 weeks early); Very Preterm (< 32 weeks’ gestation; reported as born 9 or more weeks early). Sensitivity analyses were performed to examine the impact that possible recall bias and imprecision in this measure may have had on analyses. The pattern of findings did not differ from the results presented here. See the Supplementary Information for further details of these analyses.

The ABCD developmental history questionnaire was also used to establish; maternal (biological mother) age at birth (years), sex (male or female), birth weight (lbs and ozs, converted into grams), whether their child was born by caesarian section (yes/no), or was a singleton birth (yes/no), time spent in an incubator (number of days) and time breastfed (months). The ABCD demographics questionnaire was used to establish ethno-racial identity and annual household income. In line with Barch et al. (2021), ethno-racial categories integrating caregiver-reported elements of both ethnic and racial identities of the pre-adolescents that are relevant to the US demographic, were defined as: Hispanic youth (regardless of any racial identities the caregiver also endorsed), non-Hispanic White youth, non-Hispanic Black youth, non-Hispanic Asian youth, non-Hispanic Native American/Alaska Native youth, non-Hispanic Multi-racial youth, and Additional or unknown race youth (including Native Hawaiian, Pacific Islander, Guamanian, Samoan, Other Race, no race reported). Annual household income categories were defined as; <$50,000, $50,000 to $100,000, and $100,000+.

#### Cognitive Performance

The standardised and well-validated task-based measures from the National Institute of Health Toolbox Cognition Battery (NIHTB-CB; Weintraub et al., 2014) were selected from the cognitive measures available in the ABCD dataset, to provide comprehensive assessment of cognition (Luciana et al., 2018) while minimising possible confounds and maximising comparability across studies. Executive functions measured include inhibitory control and attention (flanker task; Fan et al., 2002), working memory (list sorting working memory task; Mungas et al., 2000, 2004), task switching (dimensional change card sort task; Zelazo 2006), as well as other cognitive processes of verbal ability (picture vocabulary test; Gershon et al., 2013, 2014), processing speed (pattern comparison task; Salthouse 1992), episodic memory (picture sequence task; Dikmen et al., 2014), and reading ability (oral reading recognition task; Gershon et al., 2013, 2014). Age-corrected standardised scores (normative mean 100; SD 15) that combined accuracy and, where relevant, response time (as described in Weintraub et al., 2014) were used in the analysis.

#### White Matter Microstructure

The methods used to derive Fractional Anisotropy (FA) values have been described in full elsewhere (Hagler et al., 2019). In brief, diffusion MRI (dMRI) images were obtained across five different scanners using a 32-channel headcoil (1.7 mm isotropic voxels). Diffusion-weighted images were obtained using a multiband EPI sequence, 96 directions, with slice acceleration factor three, seven interspersed b = 0 frames, and four b-values (six directions with b = 500s/mm^2^, 15 directions with b = 1000s/mm^2^, 15 directions with b = 2000s/mm^2^, 60 directions with b = 3000s/mm^2^)^1^. T2-weighted b = 0 images were aligned to T1-weighted structural images using mutual information and were then resampled into a standard orientation with 1.7 mm isotropic resolution (for coregistration with the dMRI images).

Tract-based spatial-statistics (TBSS) were used to derive FA values. A probabilistic atlas-based method of automated segmentation of major white matter tracts was then utilised (AtlasTrack; Hagler et al., 2009). Standardised processing steps undertaken by the ABCD Data Analysis and Informatics Centre included eddy current distortion correction, motion correction, *B*0 distortion correction, and gradient nonlinearity distortion correction. FA values were calculated for the following major tracts: corpus callosum, forceps minor and major and (bilaterally): fornix, cingulate cingulum, parahippocampal cingulum, corticospinal/pyramidal tract, anterior thalamic radiations, uncinate fasciculus, inferior longitudinal fasciculus, inferior-fronto-occipital fasciculus, temporal longitudinal fasciculus, parietal longitudinal fasciculus, frontal superior corticostriate, parietal superior corticostriate, striatal inferior frontal cortex and inferior frontal superior frontal cortex. FA data was only included for participants meeting the ABCD study quality control criteria (Hagler et al., 2019).

### Statistical Analyses

#### Latent Class Analysis & Model Selection

LCA was conducted on the eight CBCL syndrome scales using dichotomous groups based on a cut-off T score of ≥ 65. Descriptive data for the CBCL scores are provided in the supplementary materials (Table S1 and Figure S1). LCA was calculated using the R package poLCA (Linzer & Lewis, 2011). We calculated models for between two and six classes and then compared models based on a range of goodness of fit criteria and interpretability of the results. Goodness of fit criteria included Bayesian Information Criteria (BIC), adjusted Bayesian Information Criterion (aBIC) and consistent Akaike Information Criterion (cAIC). Lower values of these criteria suggest better model fit. We ran 30 iterations, each with random starting points, for each of the two-to-six class models. The model estimates the probability of class membership for each individual and on this basis assigns participants to a class.

#### Associations with Class Membership

To assess whether preterm birth was associated with membership of a particular class we ran chi-square tests to determine the proportion of participants born term, Moderate-Late Preterm and Very Preterm that were allocated to each class.

The most likely class allocations were then used as the dependent variables (DVs) for a series of elastic net logistic regressions in which we assessed the contribution of cognitive and neurological factors to class membership. The elastic net regression technique is a supervised machine learning protocol which capitalises on the strengths of both ridge and lasso regression techniques to efficiently search for and select predictors whilst avoiding overfitting (Tibshirani et al., 2012). These are useful for large datasets in which choice of variables and multicollinearity between them can be problematic for traditional regression techniques (Zou & Hastie, 2005). Beta coefficients are extracted from the final model to allow comparison of the contribution of each predictor.

Six separate elastic net regressions were calculated using the CARET (Kuhn, 2008) and GLMNET (Friedman et al., 2010) packages for R (R Core Team, 2020), in order to identify the features related to membership of each class relative to the low symptom class, and each symptomatic class to the others (Class 2 versus Class 1, Class 3 versus Class 1 and Class 4 versus Class 1; Class 2 versus Class 4, Class 3 versus Class 4, and Class 2 versus Class 3). For each regression, all measures of cognitive performance and FA were input as predictors. To standardise predictor variables, all were converted to z scores (Zou & Hastie, 2005).

The dataset was randomly partitioned into training (80%) and test (20%) sets to allow us to assess how well the model could predict class membership in a separate dataset. As a much larger proportion of the sample was assigned to the low symptom class than the other classes, the training dataset, but not the test dataset, was up-sampled so that the models were trained with equal numbers of class assignments (Kuhn, 2008). In the training data we performed hyperparameter tuning, training and validation over a 10-fold cross validation framework as recommended by Kohavi, (1995). The generalisability of the best fitting model was then tested by applying it to the test data which provided the performance metrics including AUC (area under the ROC curve), precision and sensitivity.

## Results

### Sample Characteristics

The sample included in the LCA analysis consisted of 11,381 participants with a mean age of 9.48 (SD = 0.51) years, of which 5,448 (47.9%) were female. The sample characteristics are presented in Table 1. The proportion of participants born Very Preterm (1.3%) was similar to recorded USA births for 2019 (1.6%; March of Dimes, 2021), although there was a relatively greater proportion of Moderate-Late Preterm births (ABCD: 12.3% vs. USA 2019: 8.6%) and, correspondingly, a relatively smaller proportion of term births (ABCD: 86.4% vs. USA 2019: 89.8%).

**Table 1:** Sample characteristics for the full sample and by degree of prematurity

	Total (n = 11,381^a^)	Term (≤ 3 weeks early; n = 9,837 ^a^)	Moderate-Late Preterm (4 to 8 weeks early; n = 1,400 ^a^)	Very Preterm (≥ 9 weeks early; n = 144 ^a^)
Birth weight in grams, mean (SD) ^b^	3,189 (669)	3,342 (554)	2,329 (533)	1,739 (561)
Female sex, n (%)	5,448 (47.9%)	4,713 (47.9%)	665 (47.5%)	70 (48.6%)
Ethno-racial category, n (%)				
White (non-Hispanic)	5,950 (52.3%)	5,053 (51.4%)	828 (59.1%)	69 (47.9%)
Hispanic	2,324 (20.4%)	2,063 (21.0%)	228 (16.3%)	33 (22.9%)
Black (non-Hispanic)	1,688 (14.8%)	1,469 (14.9%)	190 (13.6%)	29 (20.1%)
Multi-racial (non-Hispanic)	1,036 (9.1%)	904 (9.2%)	123 (8.8%)	9 (6.3%)
Asian (non-Hispanic)	230 (2.0%)	212 (2.2%)	16 (1.1%)	2 (1.4%)
Native American/Alaska native (non-Hispanic)	34 (0.3%)	30 (0.3%)	4 (0.3%)	0
Additional & unknown	117 (1.0%)	104 (1.1%)	11 (0.8%)	2 (1.4%)
Annual household income in dollars, n (%)				
< 50,000	2,694 (23.7%)	2,380 (24.2%)	268 (19.1%)	46 (31.9%)
50,000 to 100,000	2,704 (23.8%)	2,317 (23.6%)	348 (24.9%)	39 (27.1%)
100,000 +	4,546 (39.9%)	3,885 (39.5%)	615 (43.9%)	46 (31.9%)
Not reported	1,437 (12.6%)	1,255 (12.8%)	169 (12.1%)	13 (9%)
Maternal age at birth in years, mean (SD) ^c^	29.43 (6.26)	29.32 (6.27)	30.23 (6.11)	29.13 (6.61)
Single birth, n (%) ^d^	9,249 (81.3%)	8,699 (88.4%)	488 (34.9%)	62 (43.1%)
Born by C-section, n (%) ^e^	4,283 (37.6%)	3,314 (33.7%)	870 (62.1%)	99 (68.8%)
Days in an incubator, mean (SD) ^f^	1.14 (4.75)	0.27 (1.50)	5.74 (9.48)	18.47 (16.44)
Months breastfeeding, mean (SD) ^g^	7.80 (8.54)	8.19 (8.68)	5.37 (7.11)	4.79 (6.41)

^a^ unless otherwise indicated; ^b^ not reported for 1,125 (1,045 term, 71 Moderate-Late Preterm, 9 Very Preterm) participants; ^c^ not reported for 187 (170 term, 15 Moderate-Late Preterm, 2 Very Preterm) participants; ^d^ not reported for 8 (7 term, 1 Moderate-Late Preterm) participants; ^e^ not reported for 52 (43 term, 7 Moderate-Late Preterm, 2 Very Preterm) participants; ^f^ not reported for 609 (490 term; 100 Moderate-Late Preterm; 19 Very Preterm) participants; ^g^ not reported for 345 (304 term; 34 Moderate-Late Preterm; 7 Very Preterm) participants.

### Latent Class Analysis & Model Selection

The goodness of fit statistics are summarised in Table S2 and Figure S2 within the Supplementary Information. Whilst the maximum log-likelihood provides a measure of goodness of fit, it is susceptible to overfitting, whereas information criteria such as the BIC, aBIC and cAIC attempt to avoid overfitting by penalising additional model parameters. Of these criteria, the BIC was prioritised when choosing a model due to its conservative approach in correcting for overfitting and due to its simplicity (Forster, 2000; Lin & Dayton, 1997; Nylund et al., 2007). The best fitting solution according to the BIC, aBIC and cAIC values was the four-class model. Although the likelihood ratios were slightly lower for the five-class and six-class models respectively, we selected the four-class model based on its greater parsimony and minimal loss of entropy. The average posterior probabilities of class assignment were very high (M = 0.90, SD = 0.04).

**Fig. 1:**
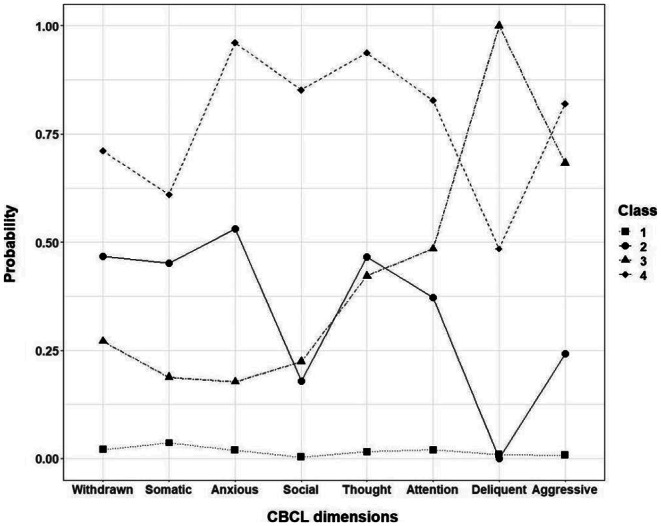
Profiles of LCA classes based on CBCL dimensions. Of 11,381 participants 88.6% were assigned to Class 1 (low symptom), 7.1% to Class 2 (predominantly internalising), 2.4% to Class 3 (predominantly externalising) and 1.9% to Class 4 (universal difficulties)

The LCA analysis of CBCL scores revealed four distinct classes (Fig. 1) 2. Participants in Class 1 (88.6%) had very low probabilities of being above threshold for any of the CBCL measures. We therefore labelled this class the ‘low symptom’ psychopathological profile and used it as the reference class for elastic net regression analyses. Class 2 was characterised by elevated probability of being above threshold for internalising behaviours, as well as thought and attention problems, and included 7.1% of the population. This class was labelled as the ‘predominantly internalising’ psychopathological profile. Class 3 was characterised by low probabilities of internalising behaviours and high probabilities of externalising behaviours, alongside increased chance of being above the cut-off for thought and attention problems. It included 2.4% of the population and was labelled as the ‘predominantly externalising’ psychopathological profile. Class 4 was characterised by high probabilities of being above threshold for all of the CBCL syndrome scales and included 1.9% of the population. Accordingly, this class was labelled as the ‘universal difficulties’ psychopathological profile.

### Analysis of PBP

To assess whether preterm birth was associated with membership of a particular class, a chi-square test was conducted. Table 2 shows the allocation of term, Moderate-Late Preterm and Very Preterm born participants to each class. The results showed no differences in the proportion of participants from each gestational category allocated to each class (χ^2^(6) = 2.15, *p* = 0.91).

**Table 2:** Allocation of participants to LCA classes split by birth at term, moderate or late preterm, or very preterm gestations

	Class 1 Low symptom	Class 2 Predominantly internalising	Class 3 Predominantly externalising	Class 4 Universal difficulties
Term, n (%)	8,707 (88.5%)	701 (7.1%)	244 (2.5%)	185 (1.9%)
Moderate-Late Preterm, n (%)	1,248 (89.1%)	99 (7.1%)	28 (2.0%)	25 (1.8%)
Very Preterm, n (%)	130 (90.3%)	10 (6.9%)	2 (1.4%)	2 (1.4%)

### Analysis of Neurocognitive Correlates

Descriptive statistics for all neurocognitive factors examined are detailed in the appendices, for the total sample and split by LCA class. 974 participants did not have MRI scan data or were removed due to quality control issues. A series of elastic net logistic regression analyses were then conducted to assess the factors that contributed to class membership.

Compared to Class 1 membership (low symptom), Class 2 membership (predominantly internalising) was predicted by 25 variables with AUC = 0.59 (95%CI = 0.58, 0.60), sensitivity = 0.55, specificity = 0.55. The beta values for the predictors are provided in Table 3. Amongst the top five predictors, membership of the predominantly internalising class was predicted by higher FA values in the inferior frontal superior frontal cortex, and lower FA values in the uncinate fasciculus, corpus callosum, frontal superior corticostriate and parietal longitudinal fasciculus.

Compared to Class 1 membership (low symptom), Class 3 membership (predominantly externalising) was predicted by 24 variables with AUC = 0.72 (95%CI = 0.71, 0.73), sensitivity = 0.66, specificity = 0.65. Amongst the top five predictors, membership of the predominantly externalising class was predicted by higher FA values in uncinate fasciculus and lower FA values in inferior longitudinal fasciculus, parietal superior corticostriate, lower reading ability and lower episodic memory ability (as measured using the oral recognition task and picture sequence task respectively).

Compared to Class 1 membership (low symptom), Class 4 membership (universal difficulties) was predicted by 24 variables with AUC = 0.69 (95%CI = 0.68, 0.70), sensitivity = 0.66, specificity = 0.64. Amongst the top five predictors, membership of the universal difficulties class was predicted by higher FA values in forceps major, parietal longitudinal fasciculus, inferior longitudinal fasciculus, forceps minor and lower FA values in the corpus callosum.

Beta values for comparisons between higher symptom classes (Class 2, Class 3 and Class 4) are in Table 4. Compared to Class 3 membership (predominantly externalising), Class 2 membership (predominantly internalising) was predicted by 25 variables (top five predictors: FA values in the parietal superior corticostriate, parahippocampal cingulum, forceps major and uncinate fasciulus, as well as verbal ability, measured using the picture vocabulary task) with AUC = 0.67 (95%CI = 0.63, 0.71), sensitivity = 0.60, specificity = 0.70.

Compared to Class 4 membership (universal difficulties), Class 2 membership (predominantly internalising) was predicted by 24 variables (top five predictors: FA values in corpus callosum, inferior fronto-occipital fasciculus, forceps major, parietal longitudinal fasciculus and forceps minor) with AUC = 0.64 (95%CI = 0.6, 0.68), sensitivity = 0.60, specificity = 0.64. Compared to Class 4 membership (universal difficulties), Class 3 membership (predominantly externalising) was predicted by 24 variables (top five predictors: FA values in corpus callosum, corticospinal pyramidal tract, inferior fronto-occipital fasciculus, forceps minor and the parietal longitudinal fasciculus) with AUC = 0.60 (95%CI = 0.53, 0.67), sensitivity = 0.61, specificity = 0.61.

To assess whether any factors were associated with class membership in a manner that differed depending on preterm birth, gestational age (in weeks) was entered into all regressions. However, although the gestational age predictor was retained in all six models it was one of the weaker predictors in each model.

**Table 3:** Cognitive and neurological factors associated with class membership (compared to the low symptom class) using elastic net regression. Table shows beta values only for those factors which were retained in the elastic net regression out of 25 possible factors (class 2: 25, class 3: 24, class 4: 24). Factors are ordered by contribution

Class 2 vs. 1 Predominantly internalising vs. low symptom		Class 3 vs. 1 Predominantly externalising vs. low symptom		Class 4 vs. 1 Universal difficulties vs. low symptom	
Predictor	Beta	Predictor	Beta	Predictor	Beta
Uncinate fasciculus	-0.354	Uncinate fasciculus	0.449	Corpus callosum	-1.482
Corpus callosum	-0.326	Inferior longitudinal fasciculus	-0.400	Forceps major	0.619
Inferior frontal superior frontal cortex	0.313	Parietal superior corticostriate	-0.378	Parietal longitudinal fasciculus	0.612
Frontal superior corticostriate	-0.293	Reading ability	-0.374	Inferior longitudinal fasciculus	0.585
Parietal longitudinal fasciculus	-0.247	Episodic memory	-0.340	Forceps minor	0.528
Parahippocampal cingulum	0.247	Corticospinal pyramidal tract	0.330	Inferior fronto occipital fasciculus	-0.446
Temporal longitudinal fasciculus	0.215	Processing speed	0.314	Working memory (EF)	-0.385
Fornix	0.177	Parahippocampal cingulum	-0.311	Temporal longitudinal fasciculus	-0.376
Striatal inferior frontal cortex	0.137	Temporal longitudinal fasciculus	0.256	Processing speed	-0.317
Corticospinal pyramidal tract	0.122	Corpus callosum	-0.246	Parietal superior corticostriate	-0.261
Anterior thalamic radiation	0.121	Cingulate cingulum	0.243	Fornix	0.225
Verbal ability	0.107	Task switching (EF)	-0.226	Corticospinal pyramidal tract	-0.178
Working memory (EF)	-0.101	Forceps major	0.225	Episodic memory	-0.172
Reading ability	− 0.083	Verbal ability	-0.216	Inferior frontal superior frontal cortex	0.169
Episodic memory	-0.079	Fornix	0.213	Gestational age	-0.151
Task switching (EF)	-0.073	Inferior fronto occipital fasciculus	-0.145	Frontal superior corticostriate	0.125
Forceps minor	-0.070	Anterior thalamic radiation	0.133	Inhibitory control & attention (EF)	0.104
Inferior fronto occipital fasciculus	0.062	Frontal superior corticostriate	0.126	Reading ability	-0.071
Cingulate cingulum	0.039	Inferior frontal superior frontal cortex	-0.125	Verbal ability	-0.070
Forceps major	0.031	Gestational age	-0.121	Parahippocampal cingulum	0.066
Parietal superior corticostriate	-0.027	Working memory (EF)	-0.094	Cingulate cingulum	-0.058
Inhibitory control & attention (EF)	0.026	Parietal longitudinal fasciculus	-0.085	Uncinate fasciculus	-0.017
Processing speed	0.025	Striatal inferior frontal cortex	0.079	Task switching (EF)	0.007
Gestational age	-0.024	Forceps minor	-0.073	Striatal inferior frontal cortex	0.004
Inferior longitudinal fasciculus	-0.015	Inhibitory control & attention (EF)	---	Anterior thalamic radiation	---

**Table 4:** Cognitive and neurological factors associated with class membership using elastic net regression. Table shows beta values only for those factors which were retained in the elastic net regression out of 25 possible factors (class 2: 25, class 3: 24, class 4: 24). Factors are ordered by contribution

Class 2 vs. 3 Predominantly internalising vs. predominantly externalising		Class 2 vs. 4 Predominantly internalising vs. Universal difficulties		Class 3 vs. 4 Predominantly externalising vs. Universal difficulties	
Predictor	Beta	Predictor	Beta	Predictor	Beta
Parietal superior corticostriate	0.388	Corpus callosum	1.464	Forceps minor	-1.45
Uncinate fasciculus	-0.363	Parietal longitudinal fasciculus	-1.058	Corpus callosum	1.29
Parahippocampal cingulum	0.347	Forceps minor	-0.743	Parietal longitudinal fasciculus	-0.89
Forceps major	-0.337	Inferior fronto occipital fasciculus	0.652	Corticospinal pyramidal tract	0.749
Verbal ability	0.330	Forceps major	-0.508	Inferior fronto occipital fasciculus	0.705
Frontal superior corticostriate	-0.329	Inferior longitudinal fasciculus	-0.471	Parahippocampal cingulum	-0.653
Cingulate cingulum	-0.30	Frontal superior corticostriate	-0.464	Inferior longitudinal fasciculus	-0.584
Episodic memory	0.270	Temporal longitudinal fasciculus	0.428	Cingulate cingulum	0.507
Anterior thalamic radiation	-0.247	Corticospinal pyramidal tract	0.379	Frontal superior corticostriate	-0.462
Striatal inferior frontal cortex	0.245	Verbal ability	0.296	Processing speed	0.374
Working memory (EF)	0.241	Processing speed	0.235	Temporal longitudinal fasciculus	0.34
Processing speed	-0.236	Parietal superior corticostriate	0.226	Verbal ability	-0.323
Inferior fronto occipital fasciculus	0.23	Working memory (EF)	0.222	Uncinate fasciculus	0.314
Forceps minor	0.134	Inhibitory control & attention (EF)	-0.19	Reading	-0.287
Reading	0.129	Inferior frontal superior frontal cortex	0.152	Forceps major	-0.286
Gestational age	0.119	Fornix	-0.127	Anterior thalamic radiation	0.233
Inferior frontal superior frontal cortex	0.11	Episodic memory	-0.111	Episodic memory	-0.223
Inhibitory control & attention (EF)	-0.103	Cingulate cingulum	-0.076	Inferior frontal superior frontal cortex	0.207
Inferior longitudinal fasciculus	-0.094	Anterior thalamic radiation	0.054	Working memory (EF)	0.165
Parietal longitudinal fasciculus	-0.09	Striatal inferior frontal cortex	-0.052	Fornix	0.117
Fornix	-0.066	Uncinate fasciculus	0.047	Inhibitory control & attention (EF)	-0.095
Temporal longitudinal fasciculus	0.06	Task switching (EF)	0.044	Gestational age	-0.048
Task switching (EF)	0.042	Gestational age	0.035	Task switching (EF)	0.04
Corticospinal pyramidal tract	0.015	Reading	0.029	Parietal superior corticostriate	-0.033
Corpus callosum	0.007	Parahippocampal cingulum	---	Striatal inferior frontal cortex	---

## Discussion

### Identification of Psychopathological Profiles

Using person-centred methods to assess how symptoms cluster within individuals, our study identified four subgroups of pre-adolescents in the ABCD study that could be distinguished by profiles of psychopathology. The four classes identified in the best fitting model reflected profiles subsequently labelled ‘low symptom’, ‘predominantly internalising’, ‘predominantly externalising’ and ‘universal difficulties’. These profiles are largely consistent with those observed in other general population samples, most of which identify at least three classes and include groups similar in nature to those identified here (Basten et al., 2013; Bianchi et al., 2017; Bonadio et al., 2016). Whilst the proportion of participants assigned to each class varies by study (presumably due to the composition of the sample and measures used), across studies the largest proportion was always assigned to the low symptom class, and the smallest proportion to the universal difficulties profile. While widespread symptomatology may affect only a small proportion of the population, understanding the neurocognitive factors associated with transdiagnostic difficulties can support future research into at-risk groups. This, in turn, may inform assessment, management and intervention. Given that additional/alternative profiles (attention/hyperactivity in Bianchi et al., 2017; severe and delinquent externalising in Bonadio et al., 2016) have only been identified in clinically-referred samples, studies should examine the sample-specificity of latent classification, and how it may vary dependent on the level of symptoms present in the sample.

The classes identified using this person-centred approach align with conclusions from variable-centred analytic models, many of which postulate a hierarchical dimensional structure of psychopathological risk, with a general factor of psychopathology (often referred to as the ‘p’ factor) reflective of shared vulnerability for any mental disorder, as well as specific vulnerability for internalising and externalising symptoms (Lynch et al., 2021). Indeed, variable-centred analyses of psychopathology in the ABCD dataset (Michelini et al., 2019; Moore et al., 2020) have also identified distinct internalising and externalising (referred to as ‘conduct’ in Moore et al., 2020) dimensions of psychopathology. However, using this person-centred analysis, we can see that our predominantly internalising and predominantly externalising profiles are also both characterised by elevated risk of problems in the CBCL attention and thought domains, suggesting that the presence of separable dimensions in datasets does not necessarily reflect how symptoms cluster in individuals. Likewise, other separable dimensions identified using variable-centred approaches, such as ADHD (Moore et al., 2020) or somatic problems (Michelini et al., 2019), did not form distinct classes in our LCA, suggesting that the risk for being above threshold on these dimensions does not commonly occur in isolation. This concurs with evidence that psychiatric disorders consistent with both internalising (depression) and externalising (conduct disorder) dimensions commonly co-occur across all ADHD subtypes (Volk et al., 2005). Yet it remains unclear whether symptoms cluster due to shared aetiologies, and accordingly, whether interventions targeted at psychopathological profiles, rather than symptom dimensions or diagnostic categories, may be effective. Moving forward, use of both person- and variable- centred approaches will be important for understanding co-occurrence and informing diagnosis and management of psychopathology across and within individuals, populations and subgroups.

### Examining the Presence of a ‘Preterm Behavioural Phenotype’

In contrast to findings from previous studies using person-centred approaches (Burnett et al., 2019; Johnson et al., 2018; Lean et al., 2019) to investigate the PBP, none of the profiles we identified were associated with overrepresentation of those born in any preterm gestational age category, and no profiles were specific to preterm birth. It could be argued that if preterm birth confers risk for a distinct psychopathological profile at this age, it should be detected in a dataset as large and diverse as the ABCD, which covers 20% of all pre-adolescents in the USA aged 9 and 10 at the time of data collection, and includes 1,400 pre-adolescents born at Moderate-Late Preterm or earlier gestations and nearly 150 born Very Preterm. However, it may well be that separable PBP profiles can only be identified in Very Preterm or Extremely Preterm samples (Burnett et al., 2019), or using indicators that focus on cognitive, as well as behavioural, functioning (Johnson et al., 2018). Recruitment of the ABCD sample was primarily school-based (Garavan et al., 2018) and those born preterm within the dataset may not represent the full spectrum of pre-adolescents born at Extremely Preterm gestations, with sample size, and perhaps representativeness, being lower for the most preterm gestations. This, or the fact that we were investigating profiles of risk associated with being above the ‘borderline’ threshold for problems in domains while sequelae following preterm birth can often remain sub-clinical, may explain why those born at preterm gestations were not overrepresented in any sub-low symptom profile.

Nevertheless, our findings contribute to a growing body of evidence that a single ‘PBP’ may not exist (Burnett et al., 2019). Indeed, other person-centred studies only partially support the presence of a PBP, with over-representation of preterm-born children in more than one sub-optimal profile (Burnett et al., 2019; Lean et al., 2019). This indicates that preterm birth may not be associated with a single cluster of symptoms. Instead, we suggest that the profiles identified in the current study reflect patterns of psychopathological difficulties that in some cases may be associated with preterm birth, but also with other factors. This conclusion is supported by the fact that gestational age at birth was only weakly associated with allocations to sub-optimal classes. The ‘predominantly internalising’ profile, whereby risk was greatest for internalising, and elevated for thought and attention problems, but lower for externalising behaviours, shows some alignment to the PBP and was the most common sub-optimal profile across all gestational categories. The key inconsistency, however, was that the most common domains of difficulty in the PBP are attention and social functioning (Fitzallen et al., 2020), while the risk for difficulty in these domains was only moderate (attention) or mild (social functioning) in the predominantly internalising class.

It is interesting to note that the four profiles identified were almost identical in nature to those produced by a four-class solution derived from a subsample comprising only those born at Moderate-Late Preterm and earlier gestations (see figure S4 in supplementary materials), so the profiles identified do not appear to have been influenced by the greater proportion of term-born children. More research is required to determine whether there are any subgroups of preterm-born pre-adolescents for whom a single PBP can be detected, be these defined on the basis of gestational category, or other factors such as age of assessment, or level of additional socio-economic risk. It may be that profiles that are distinct early on, such as the profile identified in Johnson et al. (2018), become less pronounced as maturational and compensatory processes occur during the development of preterm-born children (Wolke et al., 2019). Alternatively, it is possible that a unique PBP can only be detected in relatively homogeneous samples of children with low levels of additional socio-economic risk, an idea that is somewhat supported by the observation that the only sample in which a unique PBP was detected using latent class methods (Johnson et al., 2018) was predominantly white (~ 80%) and of low economic risk (~ 45%), with more mixed results in samples with greater diversity. Finally, it should be considered whether a preterm *cognitive*-behavioural phenotype, which includes cognitive aspects of psychopathology along with behavioural ones, might be more representative and distinct.

### Neurocognitive Correlates of Class Membership

In comparison to the low symptom class, allocation to each higher-symptom class was associated with a relatively distinct range of white matter and cognitive factors. In general, the cognitive measures were poorer predictors of class membership than FA measures, with some notable exceptions; reading ability (oral reading recognition task) and episodic memory (picture sequence task) were among the top five predictors of membership of the predominantly externalising class. In almost all cases, cognitive performance was inversely related to class membership, with the chance of being in a sub-optimal class being greater for those with lower cognitive scores, as would be expected from research linking cognitive dysfunction to a range of psychopathologies (Abela & Hankin, 2008; Snyder et al., 2015). However, for processing speed (as measured using the pattern comparison task), a positive relationship was observed with faster processing speeds associated with membership of both the predominantly externalising class, and to a lesser extent, the predominantly internalising class.

When comparing the higher-symptom classes to each other we saw some interesting patterns emerging. For example, the universal difficulties class was differentiated from all other classes by FA in a range of tracts including the corpus callosum, parietal longitudinal fasciculus, forceps minor, inferior fronto occipital fasciculus and inferior longitudinal fasciculus. FA in the forceps major was an influential predictor for membership of the internalising class relative to the universal difficulties and externalising classes, but not relative to the low symptom class. Membership of the externalising class relative to both the internalising and low symptom classes was predicted by FA in the parietal superior corticostriate and episodic memory ability (picture sequence task). Meanwhile, FA in the uncinate fasciculus appeared to differentiate between the externalising and internalising classes. Although, these analyses were exploratory in nature, the variables identified offer a starting point for developing testable hypotheses for future research, particularly around the differentiation of symptom profiles.

The importance of white matter microstructure relative to cognitive factors aligns with previous work in the same dataset linking white matter structure throughout the brain with psychopathology and executive function (Cardenas-Iniguez et al., 2020), and studies showing only small effect sizes for relations between cognitive factors and CBCL internalising/externalising scores (Thompson et al., 2019). Furthermore, the fact that white matter can be linked to psychopathological profiles suggests it is a marker that may be useful for understanding co-occurrence and reinforces the potential value in understanding mechanisms underpinning transdiagnostic risk (Castellanos-Ryan et al., 2016; Schweizer et al., 2020). That AUCs were higher for models predicting membership of externalising and universal difficulties classes than of the internalising class, suggests neurocognitive measures may be more strongly associated with these aspects of psychopathology, and is in keeping with variable-centred analyses of the ABCD dataset that show externalising and more general ‘p’ factor dimensions to be more strongly associated with poorer neurocognitive performance than internalising (Brislin et al., 2022; Moore et al., 2020; Michelini et al., 2019).

Given that higher FA values are traditionally thought to reflect increased myelination, and thus increased information processing between the connected brain regions (Fields, 2015), it may seem counterintuitive that some of the tracts showed differing positive and negative relationships between FA values and class membership. For example, relative to the low symptoms class, FA in the uncinate fasciculus was negatively associated with membership of the internalising class, but positively with membership of the predominantly externalising class, and although the corpus callosum showed a negative association with difficulties in each class, the other cross-hemispheric fibres generally showed a positive association. However, it remains highly debated as to whether increased FA values in developmental cohorts can necessarily be interpreted as greater integrity of that tract (Dodson et al., 2017; Groeschel et al., 2014). Increased FA has also been associated with ‘overconnectivity’ and the risk of psychiatric vulnerabilities such as Autistic Spectrum Disorder (Solso et al., 2016) and transdiagnostic measures of psychopathology (Hinton et al., 2019).

FA in the corpus callosum was found to inversely relate to membership of all classes reflecting increased symptom profiles, compared to the low symptom class, although it was poor at discriminating between internalising and externalising classes. In particular, this tract was the most strongly associated factor for allocation to the universal difficulties group, who had high risk of problems across all syndrome scales. This is consistent with previous studies showing that the microstructure of this region is associated with a general psychopathological risk factor (Hinton et al., 2019; Riem et al., 2019) and with overlap between ASD and ADHD (Ohta et al., 2020). It is further noteworthy that, in addition to the corpus callosum, other commissural fibres such as the fornix, and forceps major and minor, were amongst the top predictors for membership of the universal difficulties class relative to the low symptoms class, suggesting that problems with cross-hemispheric integration may underlie a variety of symptomatology.

The involvement of the fronto-striatal tracts in membership all of the higher symptom classes relative to the low symptoms class also speaks to an emerging view that variation in such tracts may underlie difficulties in goal directed behaviour, reward processing, and memory (Levitt et al., 2021), and may therefore represent a particular vulnerability to development of psychopathology. Taken together, the specificity of predictors and the direction of associations between neurocognitive factors and membership of higher symptom classes, provide further support for a shift towards a transdiagnostic approach to understanding psychopathology (Alnæs et al., 2018; Insel et al., 2010; Vanes & Dolan, 2021).

### Strengths, Limitations and Future Directions

Whilst the results and techniques used here provide new avenues for person-centred analyses of behavioural profiles, the model precision, sensitivities and effect sizes found were modest with minimum AUC values of only a little better than chance for differentiation between some models (e.g. class one vs. class two, and class four vs., class three). This is not entirely unexpected based on the likely interactions between any number of environmental and genetic variables and their effects on the relationships between brain connectivity, cognition and psychopathological profiles, and is supported by the conclusions of Owens et al. (2021) that expectations for effect sizes in such large datasets should be recalibrated. Indeed, our effect sizes align with those found in other studies using the ABCD dataset (e.g. Cardenas-Iniguez et al., 2020; Dick et al., 2021) and those from studies seeking to link genetics with behaviour and brain structure (Paulus & Thompson, 2019). However, it does mean that we should be cautious in interpretation of these models and their predictors.

The ABCD dataset provides a sample of unprecedented magnitude in which to not only use latent class approaches to assess psychopathological profiles, but also to relate these to neurocognitive functioning. This analysis used only the baseline data to assess psychopathological profiles, but with subsequent data it will become possible for future studies to generate a better understanding of whether there are different trajectories associated with different psychopathological profiles. This is of particular importance given suggestions that some psychopathological profiles may be limited to particular developmental periods (e.g. Moffitt & Caspi 2001; Moffitt et al., 2002). The factors identified here can provide a starting point for less exploratory approaches in future studies, and be used in combination with other brain imaging metrics as suggested by Figley et al. (2022).

While limited by the use of retrospective parent-reported birth data, the sensitivity analyses (see Supplementary Information) indicated that analysis of the PBP was not sensitive to concerns regarding recall bias and imprecision in the measure of gestational age for the ABCD study. However, consideration of the ABCD sampling is important when comparing our PBP findings with the wider preterm literature, particularly against findings from population-based preterm birth cohorts. Moreover, the analyses presented do not control for nested effects of family, site or scanner, nor for other variables that may affect psychopathological outcome, such as sex. Although evidence has indicated conclusions are unlikely to alter when such factors are included (e.g. controlling for site and family effects had a negligible impact on effect sizes in Owens et al., 2021; sensitivity analyses investigating the impact of scanner on brain-behaviour associations did not alter conclusions in Shen et al., 2021), studies that aim to provide conclusive answers should consider how to approach such potential confounds. It should also be noted that without controlling for these or demographic effects, the potential for bias in the selection of study sites may affect generalisability to the wider US population (Heeringa & Berglund, 2020). Future studies should consider the use of leave-one-site-out cross validation (Sripada et al., 2020). Finally, the naïve three-step analysis approach used here may have underestimated the associations between our covariates and class membership as the covariates were only introduced in the third step of the model. Future studies should consider implementing one of the corrections for this bias (e.g. Vermunt 2010).

## Conclusions

This analysis capitalised on the large ABCD dataset to add to research into the way psychopathology clusters in young people, to further examine the PBP, and to explore associations between neurocognitive factors and psychopathological profiles. Although the profiles identified were broadly consistent with findings in other young samples – with most participants allocated to the group considered to have low symptom functioning (88.6%), and smaller numbers showing increased risk for predominantly internalising problems (7.1%), predominantly externalising problems (2.4%), and universal difficulties (1.9%) – there was no evidence of a distinct phenotype associated with preterm birth. Taken together with findings from other studies, this suggests that a specific PBP may not be distinct from other profiles of psychopathology, but closer investigation is warranted. The similarity of the profiles of psychopathology identified here to prior studies, along with the relatively distinct associations with neurocognitive factors observed in relation to allocation to each sub-optimal profile, strengthen arguments that person-centred approaches to understanding psychopathology have utility for research and clinical practice. These findings highlight promising ways that use of statistical approaches suited to large datasets can deepen our understanding of the mechanisms underpinning psychopathology, and have the potential to identify neurocognitive markers of transdiagnostic risk.

## Ethics Declaration

The ABCD study received ethical approval from the University of California Institutional Review Board and our secondary analysis was approved by the host institution. The procedures used in this study adhere to the tenets of the Declaration of Helsinki.

## Electronic Supplementary Material

Below is the link to the electronic supplementary material.

Supplementary Material 1

## Footnotes

1 Full technical parameters across each of the scanners used for data acquisition is available at: https://abcdstudy.org/images/Protocol_Imaging_Sequences.pdf.

2 A Latent Profile Analysis of the continuous CBCL scores was also run which produced very similar profiles to the LCA reported here (Please see supplementary materials – Latent Profile Analysis).
