# Supplementary material for: Person-centred Approaches to Psychopathology in the ABCD Study: Phenotypes and Neurocognitive Correlates
Source: Res Child Adolesc Psychopathol. 2023 Apr 29;51(8):1195–212. doi: 10.1007/s10802-023-01065-w (PMC10368562; doi:10.1007/s10802-023-01065-w)
Supplement: Supplementary file 1 — Supplementary Material 1 [file 10802_2023_1065_MOESM1_ESM.docx]

# Supplementary Information

# **Title:** Person-centred approaches to psychopathology in the ABCD study: Phenotypes and neurocognitive correlates

# **Journal name:** Research on Child and Adolescent Psychopathology

Author names, affiliations and email address omitted for masked review

**Table S1** Descriptive statistics for CBCL scores by subscale. IQR: Inter-quartile Range.

|  | Median (IQR) | Range |
| --- | --- | --- |
| Anxious | 1 (0-4) | 0 to 26 |
| Withdrawn | 0 (0-1) | 0 to 15 |
| Somatic | 1 (0-2) | 0 to 16 |
| Social | 1 (0-2) | 0 to 18 |
| Thought | 1 (0-2) | 0 to 18 |
| Attention | 2 (0-5) | 0 to 20 |
| Delinquent | 0 (0-2) | 0 to 20 |
| Aggressive | 2 (0-5) | 0 to 36 |

**Figure S1** Frequencies of CBCL scores by subscale

**
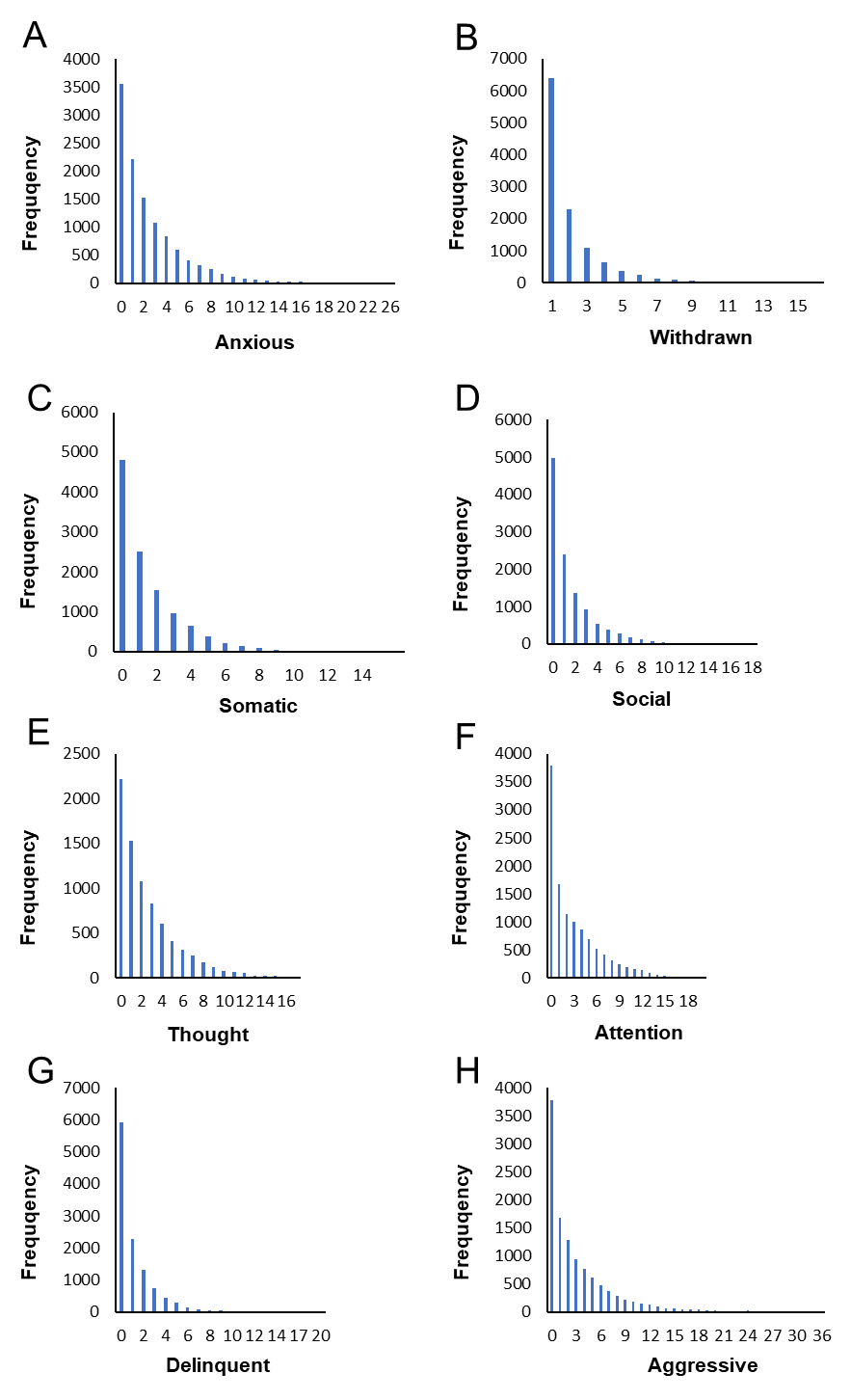
**

**Table S2** Indices of model fit

|  | **Goodness of fit criteria** | | | | | |
| --- | --- | --- | --- | --- | --- | --- |
| **Model** | **Log-likelihood** | **Resid. df** | **BIC** | **aBIC** | **cAIC** | **Entropy** |
| 2 class | -12364.92 | 238 | 24883.36 | 24829.34 | 24900.36 | --- |
| 3 class | -12236.42 | 229 | 24707.62 | 24625 | 24733.62 | 0.681 |
| 4 class | -12137.4 | 220 | 24590.87 | 24479.65 | 24625.87 | 0.733 |
| 5 class | -12110.92 | 211 | 24619.17 | 24479.35 | 24663.17 | 0.774 |
| 6 class | -12098.72 | 202 | 24676.04 | 24507.61 | 24729.04 | 0.679 |

**Fig. S2** Plots of model fit

.
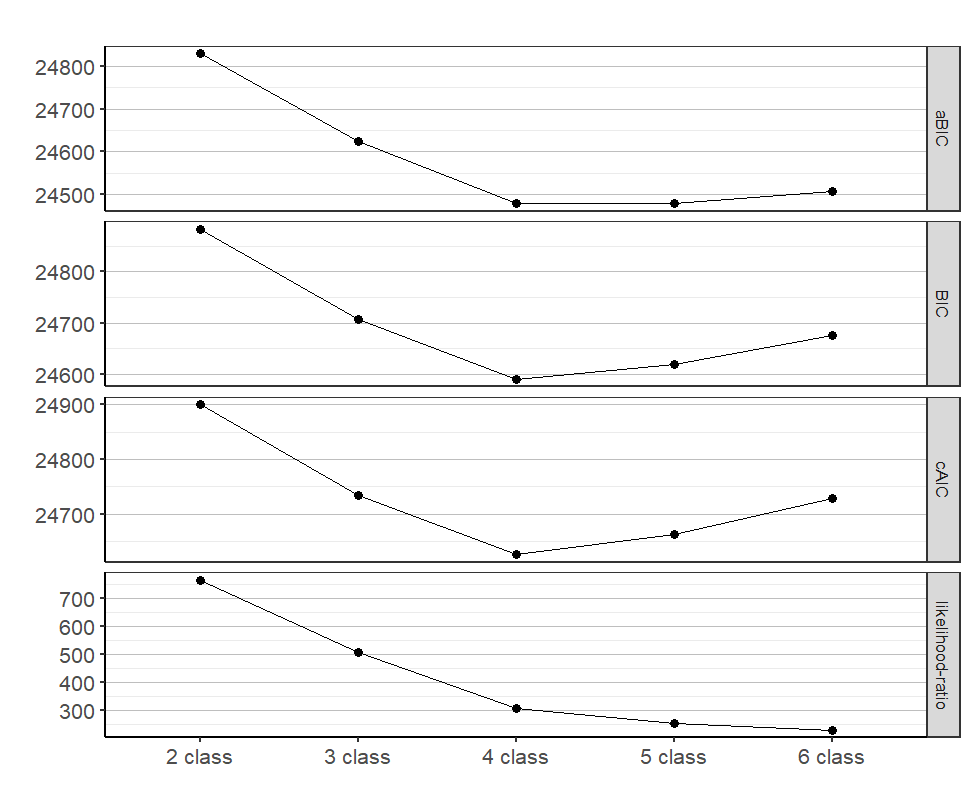


**Table S3** Age corrected scores on cognitive measures by class membership

|  | **Class 1**  Low symptom | **Class 2**  Predominantly internalising | **Class 3**  Predominantly externalising | **Class 4**  Universal difficulties | **Total** |
| --- | --- | --- | --- | --- | --- |
| Processing speed, mean (SD), n | 94.15 (21.87)  n = 9,943 | 92.47 (23.24)  n = 793 | 92.82 (23.49)  n = 270 | 88.03 (23.19)  n = 208 | 93.78 (22.09)  n = 11,632 |
| Reading ability, mean (SD), n | 103.09 (19.13)  n = 9,951 | 101.55 (19.69)  n = 793 | 94.61 (16.61)  n = 271 | 97.56 (18.96)  n = 208 | 102.55 (19.14)  n = 11,642 |
| Verbal ability, mean (SD), n | 107.17 (16.95)  n = 9,963 | 107.09 (17.48)  n = 794 | 100.09 (15.55)  n = 271 | 102.95 (18.15)  n = 209 | 106.83 (17.01)  n = 11,656 |
| Episodic memory, mean (SD), n | 101.47 (16.16)  n = 9,953 | 99.37 (15.88)  n = 794 | 94.27 (14.06)  n = 271 | 97.32 (14.57)  n = 208 | 100.99 (16.11)  n = 11,644 |
| Working memory, mean (SD), n | 101.05 (14.72)  n = 9,925 | 99.28 (14.72)  n = 792 | 95.52 (15.90)  n = 269 | 95.81 (13.31)  n = 205 | 100.58 (14.78)  n = 11,607 |
| Inhibitory control & attention, mean (SD), n | 95.71 (13.60)  n = 9,957 | 94.36 (13.38)  n = 794 | 93.09 (14.14)  n = 271 | 93.20 (14.80)  n = 209 | 95.45 (13.67)  n = 11,650 |
| Task switching, mean (SD), n | 97.13 (15.15)  n = 9,959 | 95.45 (15.29)  n = 793 | 91.90 (14.88)  n = 271 | 92.70 (14.30)  n = 208 | 96.72 (15.16)  n = 11,651 |

**Table S4** FA characteristics of the sample by class membership

|  | **Class 1**  Low symptom | **Class 2**  Predominantly internalising | **Class 3**  Predominantly externalising | **Class 4**  Universal difficulties | **Total** |
| --- | --- | --- | --- | --- | --- |
| Fornix, mean (SD), n | 0.382 (0.037)  n = 9,444 | 0.384 (0.035)  n = 733 | 0.383 (0.034)  n = 250 | 0.382 (0.036)  n = 194 | 0.382 (0.037)  n = 11,075 |
| Cingulate cingulum, mean (SD), n | 0.462 (0.053)  n = 9,444 | 0.464(0.052)  n = 733 | 0.468 (0.051)  n = 250 | 0.458 (0.052)  n = 194 | 0.462 (0.053)  n = 11,075 |
| Parahippocampal cingulum, mean (SD), n | 0.350 (0.048)  n = 9,442 | 0.353 (0.045)  n = 733 | 0.349 (0.043)  n = 250 | 0.348 (0.047)  n = 194 | 0.350 (0.048)  n = 11,073 |
| Corticospinal pyramidal tract, mean (SD), n | 0.584 (0.034)  n = 9,444 | 0.584 (0.034)  n = 733 | 0.585 (0.028)  n = 250 | 0.580 (0.034)  n = 194 | 0.583 (0.034)  n = 11,075 |
| Anterior thalamic radiation, mean (SD), n | 0.397 (0.035)  n = 9,444 | 0.40 (0.035)  n = 733 | 0.400 (0.030)  n = 250 | 0.394 (0.036)  n = 194 | 0.397 (0.035)  n = 11,075 |
| Uncinate fasiculus, mean (SD), n | 0.428 (0.040)  n = 9,443 | 0.429 (0.039)  n = 733 | 0.432 (0.038)  n = 250 | 0.426 (0.040)  n = 194 | 0.428 (0.040)  n = 11,074 |
| Inferior longitudinal fasiculus, mean (SD), n | 0.476 (0.036)  n = 9,444 | 0.477 (0.036)  n = 733 | 0.476 (0.032)  n = 250 | 0.475 (0.034)  n = 194 | 0.476 (0.036)  n = 11,075 |
| Inferior fronto occipital fasiculus, mean (SD), n | 0.485 (0.037)  n = 9,444 | 0.486 (0.037)  n = 733 | 0.487 (0.032)  n = 250 | 0.482 (0.033)  n = 194 | 0.485 (0.037)  n = 11,075 |
| Temporal longitudinal fasiculus, mean (SD), n | 0.495 (0.035)  n = 9,443 | 0.496 (0.033)  n = 733 | 0.496 (0.031)  n = 250 | 0.493 (0.034)  n = 194 | 0.495 (0.035)  n = 11,074 |
| Parietal longitudinal fasiculus, mean (SD), n | 0.465 (0.036)  n = 9,443 | 0.466 (0.035)  n = 733 | 0.466 (0.032)  n = 250 | 0.463 (0.034)  n = 194 | 0.465 (0.036)  n = 11,074 |
| Frontal superior corticostriate, mean (SD), n | 0.441 (0.030)  n = 9,444 | 0.440 (0.030)  n = 733 | 0.438 (0.029)  n = 250 | 0.436 (0.031)  n = 194 | 0.441 (0.030)  n = 11,075 |
| Parietal superior corticostriate, mean (SD), n | 0.472 (0.032)  n = 9,444 | 0.472 (0.032)  n = 733 | 0.469 (0.030)  n = 250 | 0.467(0.031)  n = 194 | 0.472 (0.032)  n = 11,075 |
| Striatal inferior frontal cortex, mean (SD), n | 0.386 (0.034)  n = 9,443 | 0.388 (0.033)  n = 733 | 0.387 (0.030)  n = 250 | 0.385 (0.035)  n = 194 | 0.386 (0.034)  n = 11,074 |
| Inferior frontal superior frontal cortex, mean (SD), n | 0.441 (0.032)  n = 9,444 | 0.442 (0.031)  n = 733 | 0.440 (0.040)  n = 250 | 0.437 (0.034)  n = 194 | 0.441 (0.032)  n = 11,075 |
| Forceps major, mean (SD), n | 0.615 (0.049)  n = 9,445 | 0.616 (0.047)  n = 733 | 0.620 (0.044)  n = 250 | 0.615 (0.048)  n = 194 | 0.615 (0.049)  n = 11,076 |
| Forceps minor, mean (SD), n | 0.517 (0.050)  n = 9,444 | 0.518 (0.049)  n = 733 | 0.519 (0.043)  n = 250 | 0.516 (0.049)  n = 194 | 0.517 (0.050)  n = 11,075 |
| Corpus callosum, mean (SD), n | 0.57 (0.039)  n = 9,445 | 0.571 (0.039)  n = 733 | 0.571 (0.035)  n = 250 | 0.567 (0.040)  n = 194 | 0.570 (0.039)  n = 11,076 |

**Fig. S3** Box plots showing distribution of scores for neurocognitive variables entered into elastic net regressions, by LCA class


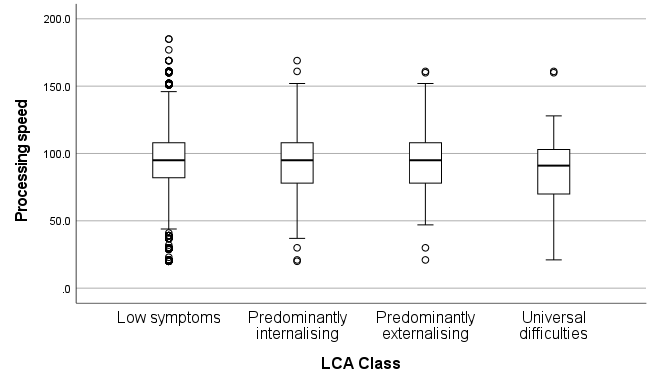

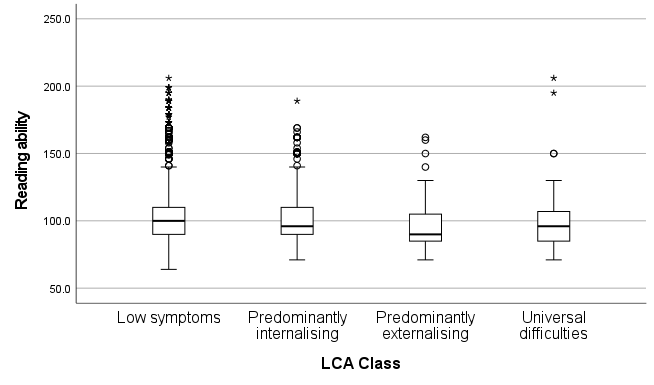


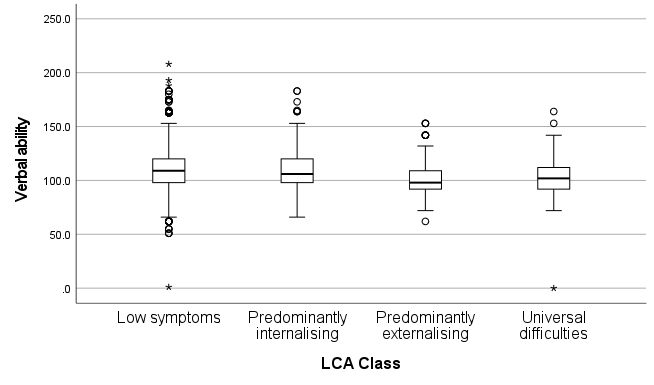

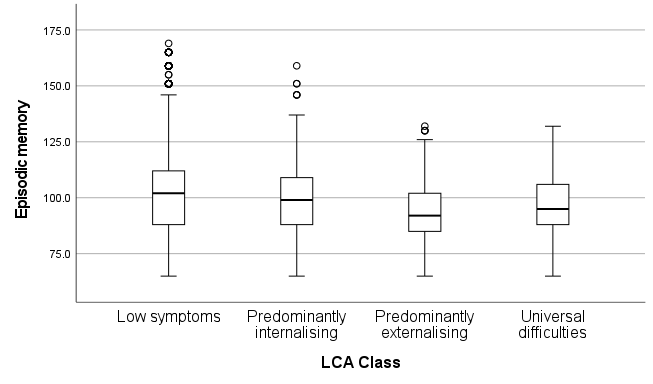


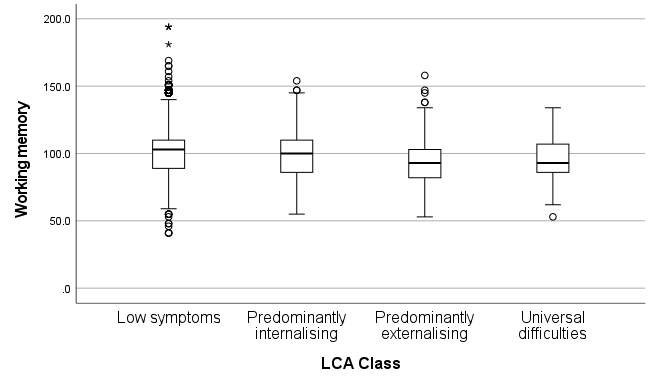

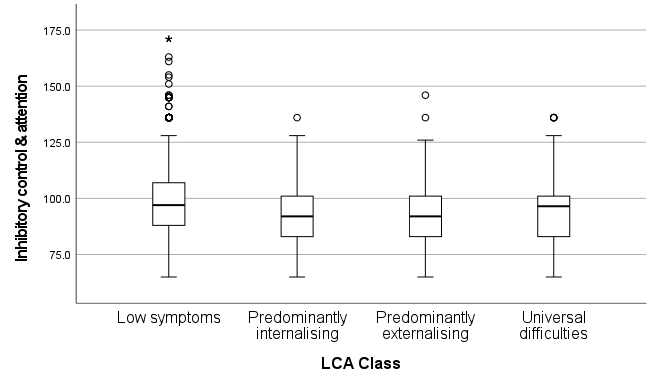


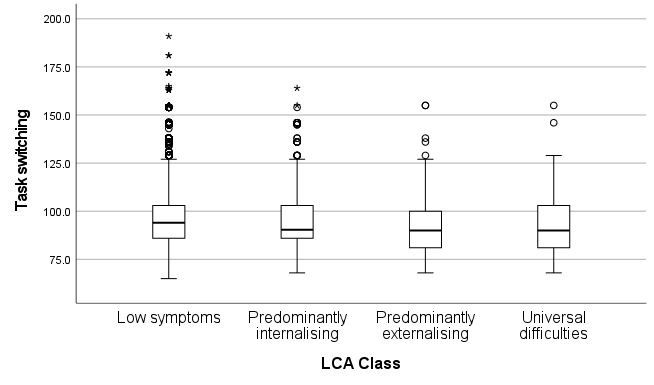

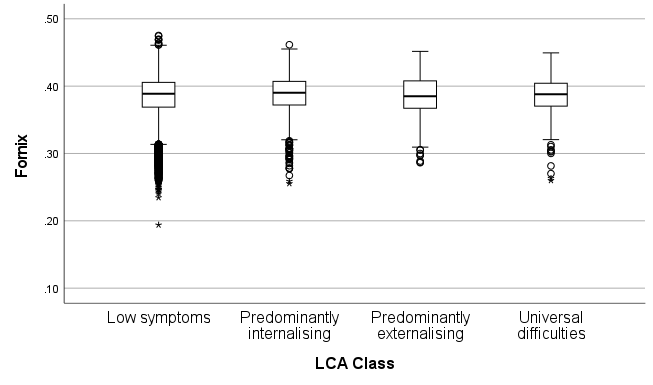


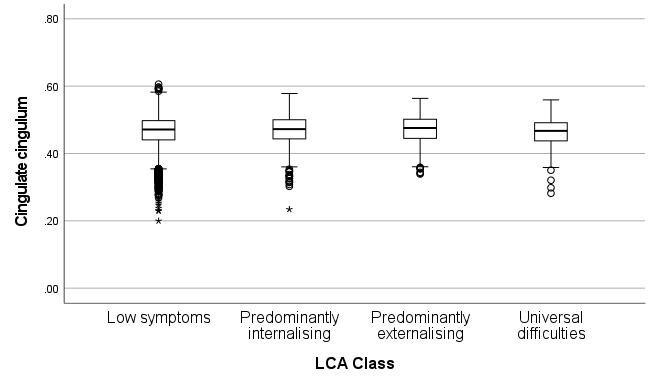

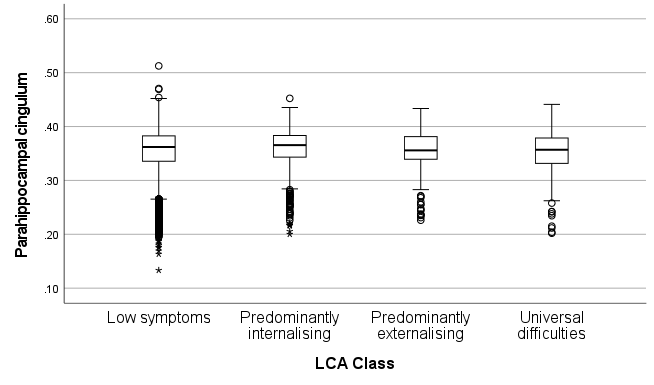


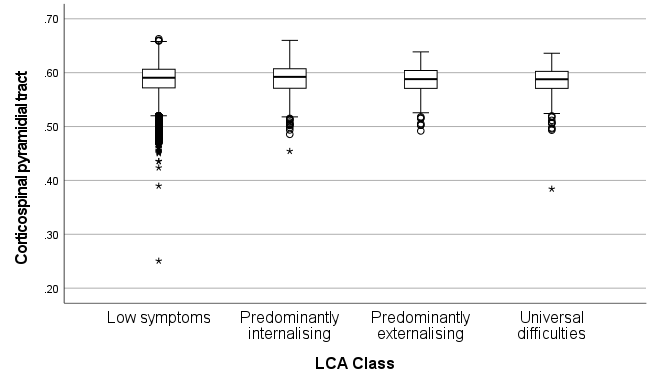

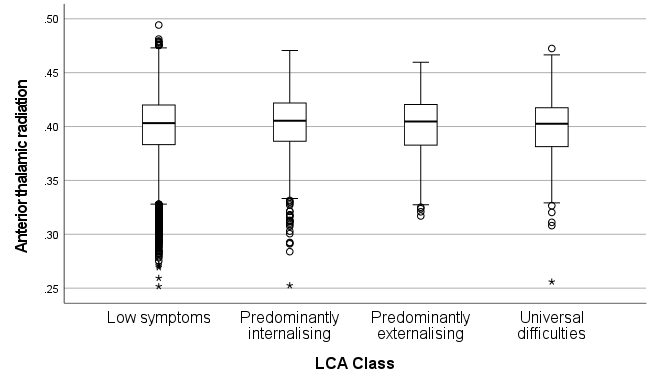


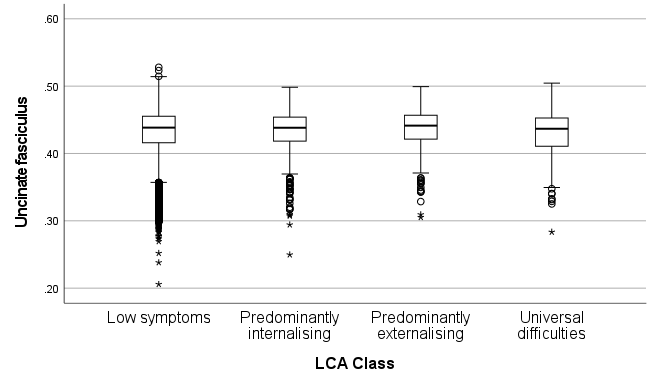

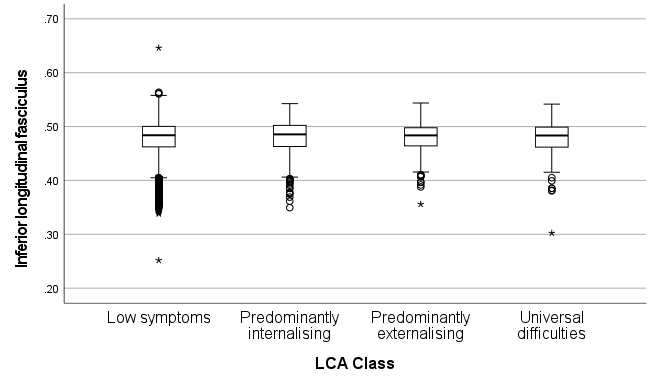


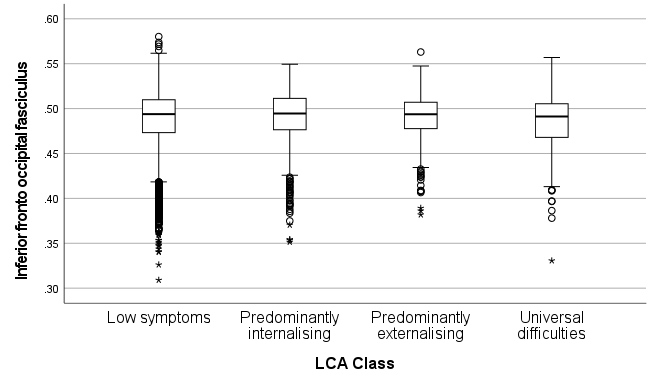

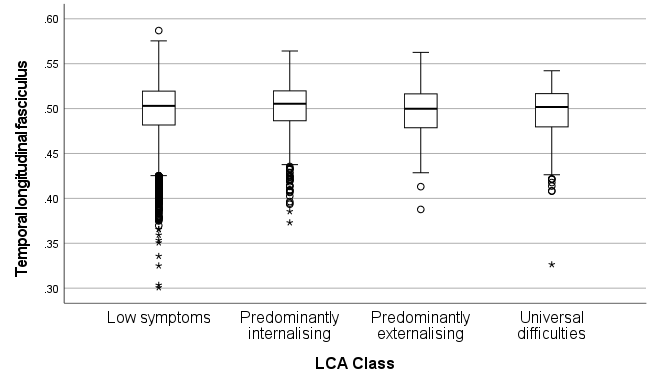


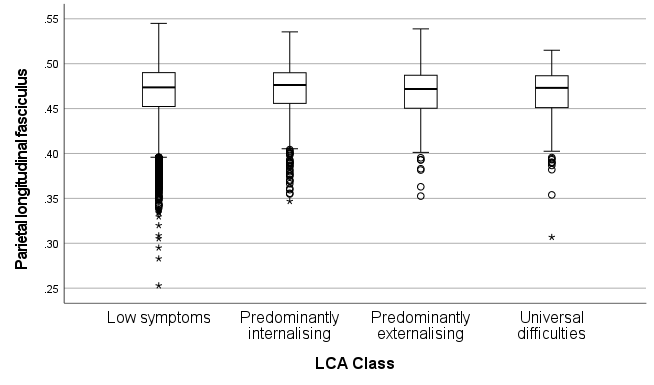

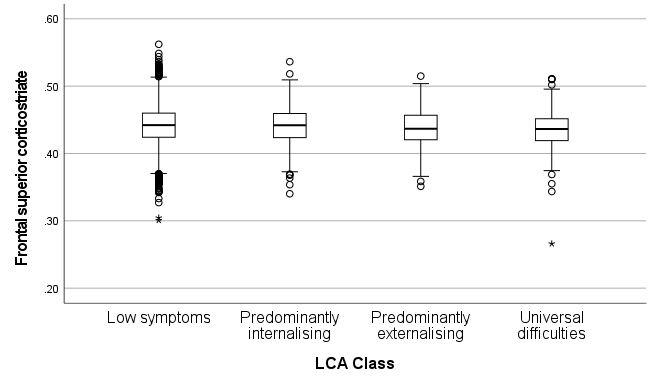


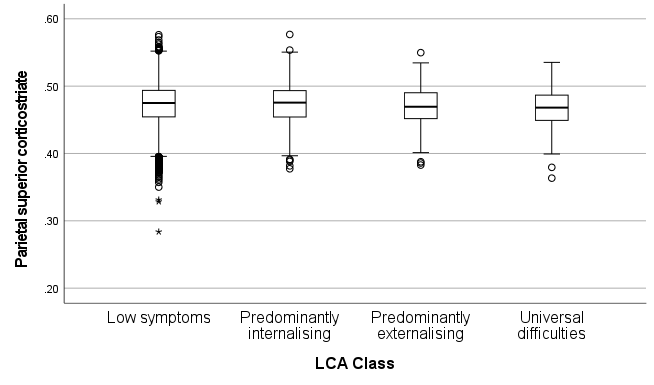

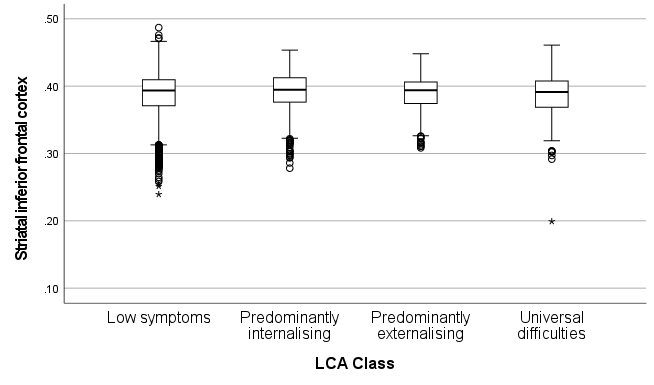


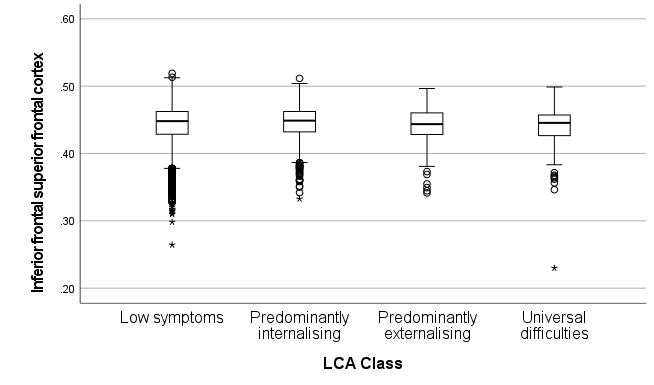

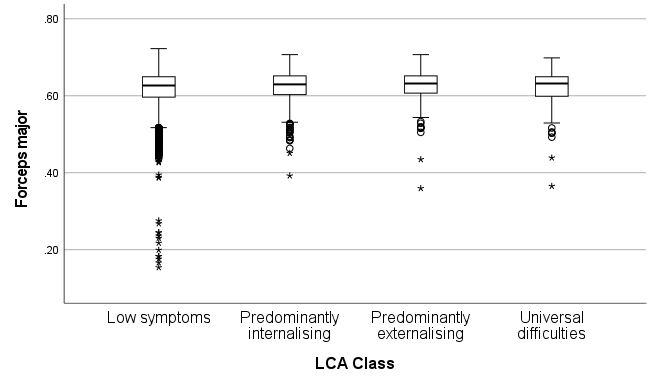


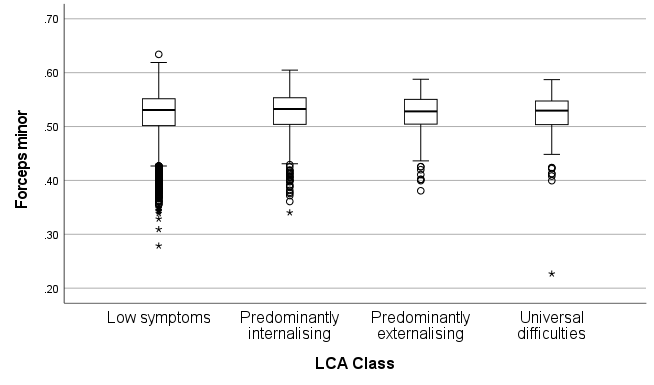

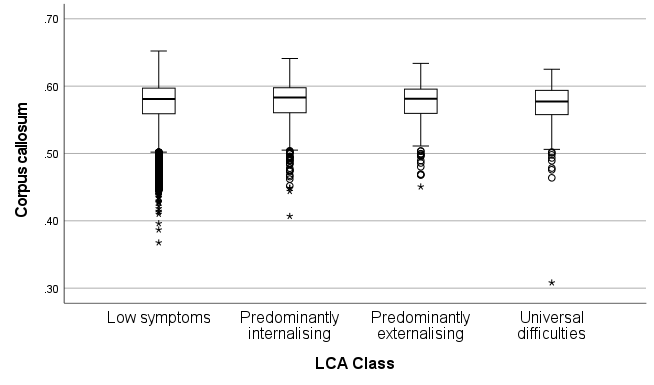


**Fig. S4** Profiles of LCA classes based on CBCL dimensions for only those born at MLPT and earlier gestations. Of 1544 participants 88.8% were assigned to Class 1 (low symptom), 7.4% to Class 2 (predominantly internalising), 2.1% to Class 3 (predominantly externalising) and 1.7% to Class 4 (universal difficulties).


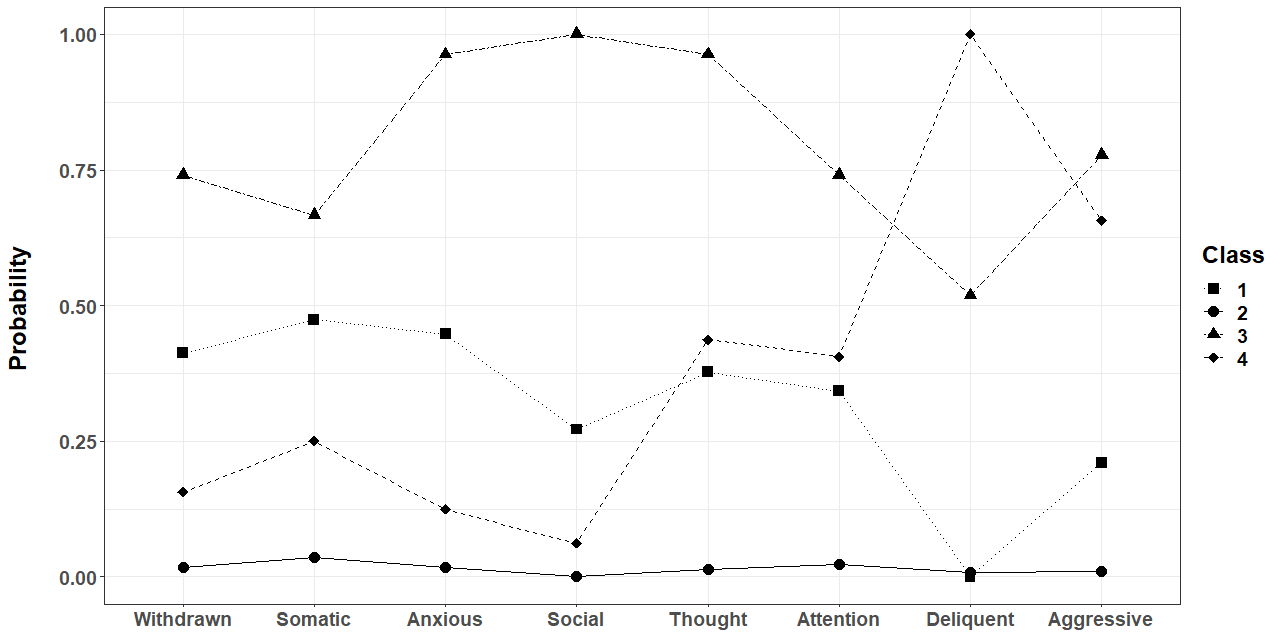


**Sensitivity analyses**

Studies have validated retrospective parent-report measures of birth data, including gestational age (Moreno-Galarraga et al., 2021; Poulsen et al., 2011), as showing high correspondence to hospital records even as late as 15 years after birth (Walton et al., 2000), indicating such measures are appropriate for use in life course studies of development (Moreno-Galarraga et al., 2021). Our main analyses of the PBP used retrospective parent recall of gestational age in weeks to categorise participants and assumed reported values were accurate, and (in the absence of precise gestational age by weeks and days) that values reported by parents corresponded to total *completed* weeks of gestation. However, we acknowledge the measure of gestational age in the ABCD dataset may be subject to issues with recall accuracy and imprecision and felt it important to assess the sensitivity of the analyses of the PBP to these assumptions.

Sensitivity analysis 1 examined the impact of excluding participants for whom the accuracy of birth data may be questionable. 1,125 participants for whom birth weight data was incomplete (i.e. a response given for only pounds or only ounces) or missing were excluded, along with a further 69 children for whom reported birth weight was outside the ranges specified by the sex-specific 3^rd^ and 97^th^ centile cut-points for the reported gestational category (Aris et al., 2019). It should be noted that although the cut-points used are based on singleton births, and the ABCD sample included twin births, no participants were excluded for implausibly low birth weight for gestational age.

Sensitivity analysis 2 examined the impact of adjusting the category boundaries for gestational age. The assumption adopted for the main analysis was that the reported gestational age reflects *completed* weeks gestation. For example, a parent report of ‘4 weeks early’ would cover births from 36^+0^ to 36^+6^, and thus a child born at 36^+5^ weeks would be accurately categorised as Moderate-Late Preterm. This interpretation is conservative in that it minimises the risk that gestational categories included children born later than the maximum cut-off for that category. However, if some parents rounded up their reports to the *nearest* week, the parent of a child born at e.g. 36^+5^, may report them as being born 3 weeks early, which corresponds to 37 completed weeks gestation, resulting in an inaccurate categorisation of term birth. In this manner, some children born close to the category boundaries may be incorrectly allocated to a category indicating a later birth. To assess the maximum impact such a bias could have, sensitivity analysis 2 used the full sample, but shifted the cut-offs used to form the gestational categories by one week. Gestational categories were redefined so that term children included those born ≤ 2 weeks early, Moderate-Late Preterm were defined as those born 3 to 7 weeks early, and Very Preterm were defined as those born 8+ weeks early.

Results for both sensitivity analyses showed the proportion of participants allocated to each latent class were almost identical to those presented in the main paper (see Table S5) and did not differ significantly by gestational age category (sensitivity analysis 1: χ^2^(6)= 1.70, *p* = 0.95 ; sensitivity analysis 2: χ^2^(6)= 3.87, *p* = 0.70).We can be confident, therefore, that these findings are not sensitive to possible concerns regarding recall accuracy and precision.

**Table S5** Allocation of participants to latent classes split by gestational age categories in sensitivity analyses 1 and 2

|  | **Class 1** Low symptoms | **Class 2** Predominantly internalising | **Class 3** Predominantly externalising | **Class 4** Universal difficulties |
| --- | --- | --- | --- | --- |
| Sensitivity analysis 1: Exclusion of those with questionable birth data | | | | |
| Term, n (%) | 7788 (88.6%) | 632 (7.2%) | 209 (2.4%) | 163 (1.9%) |
| Moderate-Late Preterm, n (%) | 1187 (89.3%) | 93 (7.0%) | 26 (2.0%) | 23 (1.7%) |
| Very Preterm, n (%) | 122 (90.4%) | 9 (6.7%) | 2 (1.5%) | 2 (1.5%) |
| Sensitivity analysis 2: Adjusted gestational category boundaries | | | | |
| Term, n (%) | 8437 (88.5%) | 684 (7.2%) | 239 (2.5%) | 178 (1.9%) |
| Moderate-Late Preterm, n (%) | 1355 (89.4%) | 105 (6.9%) | 30 (2.0%) | 26 (1.7%) |
| Very Preterm, n (%) | 293 (89.6%) | 21 (6.4%) | 5 (1.5%) | 8 (2.4%) |

**Latent Profile Analysis**

We initially ran LCA to allow comparison with relevant literature, but we have also applied Latent Profile Analyses which uses the continuous CBCL scores rather than dichotomised scores. The fit data for the LPA analysis are provided in Table S6 along with the elbow plot (Figure S5). Please note that in the elbow plot below the lines are overlapping. From the LPA analysis, however, it is unclear which model provides the best fit although arguably the elbow plot suggests that a 3 or 4-class solution may be optimal.

**Table S6** Indices of model fit

| **Model** | **AIC** | **aBIC** | **BIC** | **Entropy** | **aLMR** | **BLRT** |
| --- | --- | --- | --- | --- | --- | --- |
| 2 | 533317.5 | 533421.6 | 533501 | 0.973 | <.001 | <.001 |
| 3 | 523198.5 | 523340 | 523448.1 | 0.954 | <.001 | <.001 |
| 4 | 517698.9 | 517877.9 | 518014.6 | 0.964 | <.001 | <.001 |
| 5 | 513892.9 | 514109.4 | 514274.6 | 0.948 | 0.006 | <.001 |
| 6 | 511036.2 | 511290.1 | 511483.9 | 0.951 | 0.006 | <.001 |

**Fig. S5** Plots of model fit

As an exploratory step we next looked at the class solution for the 4-class LPA model which is plotted in Figure S6. Reassuringly, the profiles show a very similar pattern of results to that seen in the LCA analysis we have reported.

**Fig. S6** Profiles of LPA classes based on CBCL dimensions.

Finally, we looked at the allocation of preterm-born children to each of the classes using a chi-square test. As in the LCA analysis, the results showed no differences in the proportion of participants from each gestational age category allocated to each profile (χ^2^(6) = 4.82, *p* = 0.57). Given that the findings from this LPA have not altered our conclusions, and that the class solution for the LCA was more clearly defined than that for the LPA, we have opted to retain the LCA in the manuscript.

**References**

Aris, I. M., Kleinman, K. P., Belfort, M. B., Kaimal, A., & Oken, E. (2019). A 2017 US reference for singleton birth weight percentiles using obstetric estimates of gestation. *Pediatrics*, *144*(1).

Moreno-Galarraga, L., Álvarez-Zallo, N., Oliver-Olid, A., Miranda-Ferreiro, G., Martínez-González, M. Á., & Martín-Calvo, N. (2021). Parent-reported birth information: Birth weight, birth length and gestational age. Validation study in the SENDO project. *Gaceta Sanitaria*, *35*(3), 224–229. https://doi.org/10.1016/j.gaceta.2019.08.012

Poulsen, G., Kurinczuk, J. J., Wolke, D., Boyle, E. M., Field, D., Alfirevic, Z., & Quigley, M. A. (2011). Accurate reporting of expected delivery date by mothers 9 months after birth. *Journal of Clinical Epidemiology*, *64*(12), 1444–1450. https://doi.org/10.1016/j.jclinepi.2011.03.007

Walton, K. A., Murray, L. J., Gallagher, A. M., Cran, G. W., Savage, M. J., & Boreham, C. (2000). Parental recall of birthweight: A good proxy for recorded birthweight? *European Journal of Epidemiology*, *16*(9), 793–796. https://doi.org/10.1023/A:1007625030509
